# Cross-cultural adaptation of the State Behavioral Scale to Brazilian Portuguese

**DOI:** 10.62675/2965-2774.20250183

**Published:** 2025-03-14

**Authors:** Janaína Santana Dantas, Martha Moreira Cavalcante Castro, Carolina Villa Nova Aguiar

**Affiliations:** 1 Hospital Universitário Professor Edgard Santos Universidade Federal da Bahia Salvador BA Brazil Hospital Universitário Professor Edgard Santos, Universidade Federal da Bahia - Salvador (BA), Brazil.; 2 Universidade Federal da Bahia Salvador BA Brazil Universidade Federal da Bahia - Salvador (BA), Brazil.; 3 Escola Bahiana de Medicina e Saúde Pública Salvador BA Brazil Escola Bahiana de Medicina e Saúde Pública - Salvador (BA), Brazil.

**Keywords:** State Behavioral Scale, Pediatric intensive care units, Artificial respiration, Cross-cultural adaptation, Surveys and questionnaires, Brazil

## Abstract

**Objective:**

To perform a cross-cultural adaptation of the State Behavioral Scale to Brazilian Portuguese, assess its psychometric quality and use the scale to evaluate the level of sedation of patients on mechanical ventilation in the pediatric intensive care unit of a tertiary care hospital.

**Methods:**

After receiving authorization by the main author, the State Behavioral Scale was adapted according to the following steps: translation of the original version into Portuguese; synthesis of the Portuguese versions; evaluation by a committee of judges; reverse translation by native speakers of the source language; synthesis of retroversions; pretest; and evaluation of psychometric quality.

**Results:**

The adapted scale was administered to 20 patients by four evaluators, who performed daily evaluations in pairs simultaneously and independently. The intraclass correlation coefficient was 0.939 (p < 0.001) for the State Behavioral Scale and 0.976 (p < 0.001) for the COMFORT-B scale. The two scales were strongly correlated, with Spearman coefficients ranging from 0.884 to 0.908 (p < 0.001). In the study sample, most children (n = 43 observations; 48.9%) had scores of -1 (responsive to light touch or voice) or 0 (awake and able to calm down), which corresponded to light sedation.

**Conclusion:**

The translated and adapted version of the State Behavioral Scale showed high interrater agreement and high correlation with the COMFORT-B scale. The application of the scale showed an adequate level of sedation in most patients.

## INTRODUCTION

The pediatric intensive care unit (ICU) is an environment with extensive technological advancements that provide greater survival for patients. However, invasive therapeutic interventions, continuous monitoring and underlying pathological processes can cause painful or unpleasant experiences for the child, which can lead to agitation, fear and anxiety.^1,2^

Other factors, such as the presence of strangers, noise from numerous pieces of equipment, disruption of the sleep‒wake cycle and separation from family members, can cause emotional distress, anxiety and insomnia. In this context, the need for sedatives and analgesics in intensive care is undeniable.^2^

The goal of sedative use is to reduce the anxiety and agitation that affect a child when faced with a hostile environment and numerous manipulations, whereas the aim of analgesic use is to reduce the pain caused by the procedures or even the underlying disease.^3^ Sedoanalgesia also increases patient safety by preventing falls and accidental removal of devices necessary for the maintenance of life, such as endotracheal tubes, feeding tubes, and venous and arterial catheters.^4,5^

Sedation covers a spectrum from minimal sedation to general anesthesia.^6^ Notably, the sedative needs of each patient change constantly depending on the nature and course of the disease, as well as the interactions of the sedative with other therapies. Achieving an optimal and light level of sedation is ideal to avoid overdosing or underdosing, which are each associated with related adverse events.^7,8^

Therefore, continuous reassessment of the level of sedation is essential. Several good clinical practice recommendations emphasize the need for goal-oriented therapy in the administration of sedatives and advocate for this approach as the standard of care in ICUs, due to the impact of the use of these medications on the discontinuation of ventilatory support and length of ICU stay.^8^

The use of an instrument to measure the level of sedation is essential to follow the recommendations for goal-guided therapy.^5,9^ Although the clinical and subjective judgment of caregivers is important, the application of an objective, practical and easy-to-perform instrument is necessary to ensure greater accuracy in the evaluation.^10,11^

There is no gold standard scale for sedation assessment in pediatrics; each hospital implements the scale that is considered the most appropriate for the population and its staff. The scales most commonly used in pediatric ICUs are the COMFORT scale (37%), State Behavioral Scale (24%) and Ramsay Sedation Scale (18%).^12^

The COMFORT scale was first described in 1992 and was adapted for critically ill pediatric patients.^13^ This scale includes both behavioral and physiological parameters (heart rate and blood pressure), the latter of which are usually controlled in an intensive care environment; therefore, in 2005, the COMFORT-Behavior scale (COMFORT-B), containing only the behavioral variables, was validated as an alternative to the original scale.^14^ In 2008, Amoretti et al. translated the COMFORT-B into Portuguese and validated the translation, which represents the first validation of a sedation scale for the Brazilian pediatric population.^15^

The Ramsay scale is a simple, intuitive scale with six levels of sedation. However, these levels are not mutually exclusive or clearly defined, and the scale only describes one level of agitation.^16,17^

The Richmond Agitation-Sedation Scale (RASS) is a specific scale for the evaluation of sedation and agitation; it is composed of ten items and three well-defined steps. In 2021, Massaud-Ribeiro et al. performed the cross-cultural translation of the RASS into Brazilian Portuguese. The RASS is easy to administer and less extensive than the COMFORT-B.^17^

The State Behavioral Scale is widely used in the United States;^18^ however, a Portuguese version has not yet been validated. Because this scale is an objective scale that is quickly performed and easily understood compared with existing scales, greater adherence to the use of the State Behavioral Scale by all pediatric intensive care providers in Brazil is expected.

Therefore, the aim of the present study was to perform a cross-cultural adaptation of the State Behavioral Scale into Brazilian Portuguese, assess its psychometric quality and use the translated scale to evaluate the level of sedation of mechanically ventilated patients in the pediatric intensive care unit of a tertiary hospital.

## METHODS

This study was a methodological study of the translation, cross-cultural adaptation and evaluation of the psychometric quality of the State Behavioral Scale in Brazilian Portuguese. The procedures adopted in this study followed the model proposed by Borsa et al.: obtaining the permission of the lead author, translation and agreement, back translation and agreement, analysis by a committee of experts, pretest in a target population, and review and construction of the final version.^19^

The State Behavioral Scale was originally constructed in English by Curley et al. to systematically describe the sedation‒agitation continuum in pediatric patients on mechanical ventilation support. This scale includes eight dimensions, namely, respiratory drive, response to ventilation, coughing, best response to stimulation, attentiveness to care provider, tolerance to care, consolability and movement after consoled; each dimension has 3–5 levels, and the scale defines six behavioral states. The operational definitions of the scale are as follows: **sedation**, a calm and tranquil state that relieves anxiety and arousal; **agitation**, excitation accompanied by increased motor activity; **attention**, the ability to open one’s eyes and observe one’s surroundings; **responsiveness**, the ability to open one’s eyes or raise one’s eyebrows, turn one’s head toward a stimulus, or move one’s limbs; and **discomfort**, a sudden increase in heart rate or blood pressure and/or a decrease in oxygen saturation or increased movement.^16^

The adaptation protocol was performed as follows: after authorization from the original author, the scale was translated by two independent researchers. A third party performed a comparative analysis of the two translations and proposed a synthesis, which was discussed by the research team. Through consensus, the research team arrived at the Portuguese version of the scale, which was submitted for evaluation by a committee of judges.

The analysis by the expert committee was performed by forwarding a form with the translated version of the scale to 10 judges/experts in pediatric intensive care. Each expert was asked to evaluate the 40 items of the scale on a Likert-type response scale with four options (1, not relevant or not representative; 2, item needs major revision to be representative; 3, item needs small revision to be representative; 4, relevant or representative item). In the form, the judges could also make suggestions for each item if they considered it necessary.

For data analysis, the content validity indices (CVIs) of each item were calculated according to the following formula:


$$\mathrm{CVI}=\frac{\text{ Number of judges who scored item }3\text{ or }4}{\text{ Total number of judges }}$$

The items with CVIs below 0.78 were reanalyzed by the researchers; in addition, the suggestions were reviewed, and changes were made to better meet the needs of the scale.^20^ The items that were changed were evaluated again by the same experts, and new CVI calculations were performed.

After the modifications derived from the expert analysis, the final Portuguese version was developed, which was translated back into English by two independent translators who were native English speakers and fluent in Portuguese. The translators were not familiar with the concepts addressed in the scale or the original scale in English. The two versions of the back-translation were analyzed by the researchers, who produced a third version (synthesis) and sent it to the original author of the State Behavioral Scale in English for analysis.

The study was approved by the Research Ethics Committee of the Bahia School of Medicine and Public Health (EBMSP) in accordance with the Brazilian Ethical Resolutions and, in particular, CNS Resolution 510/2016 (approval number: 5,160,912).

The adapted scale was evaluated from October 2021 to February 2022 in two ICUs of the *Hospital Martagão Gesteira*/Salvador-BA, the largest exclusively pediatric hospital in the North and Northeast Regions. The inclusion criteria for the study participants were children aged 6 weeks to 6 years who were on mechanical ventilation and receiving sedatives. Children who had received neuromuscular blockade or had neuromuscular diseases; patients hospitalized for pain assessment only; and patients considered physiologically unstable (children who had any increase in ventilation or vasopressor support in the previous two hours) were excluded.

The adapted version of the State Behavioral Scale and the COMFORT-B scale were administered by a team of four physicians who worked in pairs to perform the observations and administered the scales both simultaneously and independently. Patients were classified according to the degree of sedation on the basis of the scores on the translated version of the State Behavioral Scale and the COMFORT-B scale.

The data were stored and analyzed using the IBM Statistical Package for the Social Sciences (SPSS), version 20.0 (IBM Corp., Armonk, NY, USA). For the sociodemographic data, absolute and relative frequencies are used to describe the qualitative variables, and medians with interquartile ranges (IQRs) are used to describe the quantitative variables. The reproducibility of the scales was assessed using the intraclass correlation coefficient (ICC); an ICC greater than 0.75 was considered excellent, an ICC between 0.4 and 0.75 was moderate, and an ICC less than 0.4 was poor.^21^ The ICC is usually considered equivalent to the square-weighted kappa value used in the original study.^22,23^

Convergent validity was calculated using Spearman’s correlation coefficient, which assessed the level of relationship or correspondence between the results obtained using the translated version of the State Behavioral Scale and those obtained with the COMFORT-B scale. The correlation was considered high for a coefficient greater than 0.6, moderate for a coefficient between 0.4 and 0.6, and low for a coefficient less than 0.4.^24^

## RESULTS

The 40 items of the scale were analyzed by a committee of judges composed of four (40%) physicians, four (40%) physical therapists and two (20%) nurses. The CVI of each item was calculated, and three items had a CVI equal to 0.7, as shown in table 1.

**Table 1:** Content validity index, modifications and considerations for each item

Descrição: Não responsivo	IVC 1	Modificação depois da 1ª análise dos juízes	IVC depois das modificações (considerações)
*Sem esforço respiratório espontâneo*	*1*	*Sem necessidade de modificação*	-
*Sem tosse ou tosse apenas com aspiração*	*0,9*	*Sem necessidade de modificação*	-
*Sem resposta a estímulos dolorosos*	*1*	*Sem necessidade de modificação*	-
*Incapaz de prestar atenção ao provedor de cuidados*	*0,9*	*Incapaz de prestar atenção ao provedor de cuidados*	-
*Não reage a nenhum procedimento (inclusive doloroso)*	*0,9*	*Sem necessidade de modificação*	-
*Não se move*	*0,9*	*Sem necessidade de modificação*	-
**Descrição: Responsivo a estímulos dolorosos**	**IVC 1**	**Modificação depois da 1ª análise dos juízes**	**IVC depois das modificações (considerações)**
*Respiração espontânea, mas ainda com suporte ventilatório*	*1*	*Sem necessidade de modificação*	-
*Tosse durante aspiração ou reposicionamento*	*0,9*	*Sem necessidade de modificação*	-
*Responde ao estímulo doloroso*	*0,9*	*Sem necessidade de modificação*	-
*Incapaz de prestar atenção ao provedor de cuidados*	*0,9*	*Sem necessidade de modificação*	-
*Reage a um procedimento doloroso*	*1*	*Sem necessidade de modificação*	-
*Não se move/Apresenta movimento ocasional de membros ou mudança de posição*	*1*	*Sem necessidade de modificação*	-
**Descrição: Responsivo ao toque leve ou à voz**	**IVC 1**	**Modificação depois da 1ª análise dos juízes**	**IVC depois das modificações (considerações)**
*Respiração espontânea, mas ineficaz sem o suporte ventilatório*	*0,9*	*Sem necessidade de modificação*	-
*Tosse durante aspiração ou reposicionamento*	*0,8*	*Sem necessidade de modificação*	-
*Resposta ao toque ou voz*	*0,9*	*Sem necessidade de modificação*	-
*Capaz de prestar atenção apenas durante a estimulação*	*1*	*Sem necessidade de modificação*	-
*Desconforta com procedimentos*	*0,9*	*Sem necessidade de modificação*	-
*Capaz de se acalmar com toque ou voz reconfortante quando o estímulo é removido*	*1*	*Sem necessidade de modificação*	-
*Apresenta movimento ocasional de membros ou mudança de posição*	*1*	*Sem necessidade de modificação*	-
**Descrição: Acordado e capaz de se acalmar**	**IVC 1**	**Modificação depois da 1ª análise dos juízes**	**IVC depois das modificações (considerações)**
*Respiração espontânea e efetiva*	*1*	*Sem necessidade de modificação*	-
*Tosse quando reposicionado / Tosse espontânea ocasional*	*0,8*	*Sem necessidade de modificação*	-
*Reage à voz / Nenhum estímulo externo é necessário para obter resposta*	*1*	*Sem necessidade de modificação*	-
*Presta atenção espontaneamente ao provedor de cuidados*	*1*	*Sem necessidade de modificação*	-
*Desconforta com procedimentos*	*0,8*	*Sem necessidade de modificação*	-
*Capaz de se acalmar com toque ou voz reconfortante quando o estímulo é removido*	*1*	*Sem necessidade de modificação*	-
*Apresenta movimento ocasional de membros ou mudança de posição/Movimentos aumentados (inquieto, contorce-se)*	*0,9*	*Sem necessidade de modificação*	-
**Descrição: Inquieto e difícil de acalmar**	**IVC 1**	**Modificação depois da 1ª análise dos juízes**	**IVC depois das modificações (considerações)**
*Respiração eficaz espontânea / com dificuldade de respirar com ventilador*	*1*	*Sem necessidade de modificação*	*-*
*Tosse espontânea ocasional*	*0,7*	*Tosse espontaneamente com frequência*	*1 (Os peritos entendem que se o paciente está inquieto, ele tosse com frequência, incomodado principalmente com a presença do tubo endotraqueal em via aérea)*
*Responde à voz/ Não necessita de estímulo externo para obter resposta*	*0,8*	*Sem necessidade de modificação*	*-*
*Se afasta / Espontaneamente presta atenção ao provedor de cuidados*	*0,7*	*Percebe espontaneamente a presença do provedor de cuidados e tende a se afastar (esquivar)*	*0,9 (A mudança de expressão de “prestar atenção” para “percebe a presença” trouxe melhora do índice), além de acrescentar mais elementos descritivos como a palavra “esquivar” entre parênteses)*
*Frequentemente inseguro*	*0,8*	*Frequentemente inseguro (morde o tubo endotraqueal, puxa cateteres, não pode ficar sozinho)*	*1 (Apesar de ter atingido a nota de corte, como havia sido motivo de várias sugestões com o questionamento de que a palavra “inseguro” não estava bem definida, como estava no nível* **Agitado***, sendo apenas acrescentado o que seria inseguro ipsis litteris ao que já existia no nível* **Agitado***)*
*Não se acalma consistentemente apesar da tentativa de 5 minutos / incapaz de consolar*	*1*	*Sem necessidade de modificação*	-
*Movimento aumentado (inquieto, contorcendo-se)*	*1*	*Sem necessidade de modificação*	-
**Descrição: Agitado**	**IVC 1**	**Modificação depois da 1ª análise dos juízes**	**IVC depois das modificações (considerações)**
*Pode apresentar dificuldade em respirar com o ventilador*	*1*	*Sem necessidade de modificação*	-
*Tosse espontaneamente*	*0,9*	*Sem necessidade de modificação*	-
*Nenhum estímulo externo é necessário para obter resposta*	*1*	*Sem necessidade de modificação*	-
*Presta atenção espontaneamente ao provedor de cuidados*	*0,7*	*Percebe espontaneamente a presença do provedor de cuidados e tende a se afastar (esquivar)*	*0,9 (Novamente, a mudança da expressão “presta atenção” para “percebe a presença” trouxe melhora do índice), além de acrescentar mais elementos descritivos como a palavra “esquivar” entre parênteses. Para os peritos, não há diferença dessa dimensão para o nível* **Inquieto e Difícil de acalmar***)*
*Inseguro (morde o tubo endotraqueal, puxa cateteres, não pode ficar sozinho)*	*1*	*Sem necessidade de modificação*	-
*Incapaz de ser consolado*	*1*	*Sem necessidade de modificação*	-
*Movimento aumentado (inquieto, contorcendo-se ou debatendo-se de um lado para o outro, chutando as pernas)*	*1*	*Sem necessidade de modificação*	-

Translated and adapted from Source: Curley MA, Harris SK, Fraser KA, Johnson RA, Arnold JH. State Behavioral Scale: a sedation assessment instrument for infants and young children supported on mechanical ventilation. Pediatr Crit Care Med. 2006;7(2):107-14.

IVC - índice de validade de conteúdo.

The three items that were below the cutoff value (0.78) and one additional item that, despite having a CVI of 0.8, aroused semantic doubts were analyzed among the researchers, and the expert suggestions were evaluated. The changes were made, and the items were reanalyzed by the same 10 experts. After the changes described in table 1, the four items obtained scores above the cutoff value. Table 1 also shows the modifications submitted for the second expert analysis and the new CVIs and considerations.

After evaluation by the committee of experts, the final version was sent for back translation. Two versions in English were obtained, and their synthesis was forwarded to the original author, who made some suggestions and approved the version.

The pretest was performed in two ICUs with clinical-surgical profiles in a convenience sample of 20 patients, resulting in a total of 88 observations during the study period. The sample consisted of children with a median age of 5.5 months (IQR = 2.0–12.0), with an equal distribution between boys and girls, as shown in table 2. The reason for ICU admission was distributed into four categories (oncological, respiratory, cardiac postoperative period and sepsis); 65% of the patients were cardiac postoperative patients who had a median of 12 days of mechanical ventilation and 13 days of hospitalization at the time of observation.

**Table 2:** Demographic and clinical characteristics of the 20 study participants

Characteristics	n (%)	Median (IQR 25-75)
Age (months)		5.50 (2.00 - 12.00)
Sex		
Female	10 (50)	
Male	10 (50)	
Primary diagnosis		
Oncological	1 (5)	
Respiratory	3 (15)	
Cardiac postoperative	13 (65)	
Sepsis	3 (15)	
Time on mechanical ventilation (days)		12.0 (3.0 - 15.0)
Length of stay (days)		13.0 (4 - 21.7)

IQR - interquartile range.

**Figure 1:**
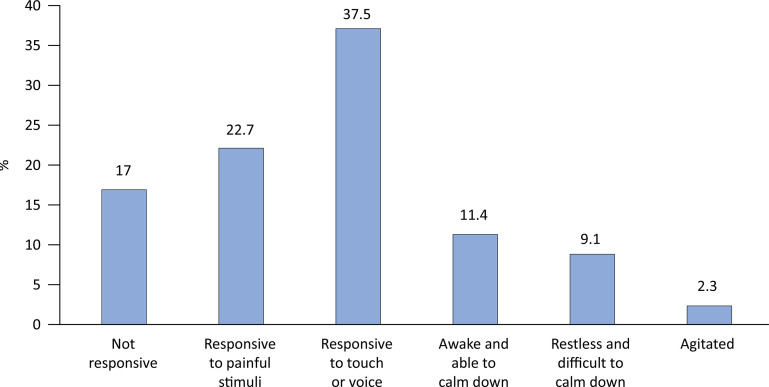
Classification of participants according to the State Behavioral Scale adapted for Brazil.

Figure 1 shows the frequency of classification of patients according to the results of the adapted State Behavioral Scale: 48.9% of the patients had scores of -1 (37.5%; responsive to light touch or voice) or 0 (11.4%; awake and able to calm down), which correspond to light sedation.

The State Behavioral Scale and COMFORT-B scale had excellent intraclass correlations (0.939 and 0.976, respectively), as shown in table 3.

**Table 3:** Intraclass correlation coefficients for the State Behavioral Scale and COMFORT-B scale

Ownership	State Behavioral Scale	p value	COMFORT-B	p value
Reproducibility (ICC)	0.939	0.001	0.976	0.001

ICC - intraclass correlation coefficient.

To verify the relationship between the State Behavioral Scale and the COMFORT-B scale, Spearman’s correlation was performed as shown in table 4. All the results indicated overlap between the two scales, indicating that both scales assess the same construct.

**Table 4:** Spearman correlation index between the State Behavioral Scale and COMFORT-B scale and between raters 1 and 2

	COMFORT-B (evaluator 1)		COMFORT-B (evaluator 2)	
	r _s_	p value	r _s_	p value
State Behavioral Scale (evaluator 1)	0.900	0.001	0.884	0.001
State Behavioral Scale (evaluator 2)	0.908	0.001	0.889	0.001

## DISCUSSION

This study is the first translation and cross-cultural adaptation of the State Behavioral Scale from English to Brazilian Portuguese. The linguistic and semantic equivalences between the original scale and the Brazilian Portuguese version were satisfactory.

The COMFORT-B scale is the most widely used scale worldwide and was the first pediatric scale adapted to Brazilian Portuguese.^12,15^ Due to its relevance and wide use, we chose to correlate the State Behavioral Scale with the COMFORT-B scale. According to the present study, the scales exhibit an excellent correlation; the State Behavioral Scale has the advantage of being more practical to administer, which may explain its predominance in American pediatric ICUs.^12^

The COMFORT-B scale is unanimously considered complex but important in the pediatric intensive care setting. In 2014, Mendes et al. compared the COMFORT-B scale with the Ramsay scale; despite a good correlation between the scales, the time spent in the application of the COMFORT-B scale was significantly longer,^25^ which may have interfered with the care teams’ adherence to its use.

Another advantage of the State Behavioral Scale is that it is exclusively used to assess sedation, as the COMFORT-B scale also includes a pain assessment.^15^ Another significant difference between the scales is that the State Behavioral Scale includes three domains, attentiveness to the caregiver, tolerance of care and consolability, that are not included in the COMFORT-B scale.

Notably, many physicians still do not adopt a sedation protocol, which complicates evaluation and therapeutic practice. In an international study, Kudchadkar et al. reported that although 70% of the respondents stated that they worked in hospitals with sedation protocols, only 42% used these protocols routinely to determine the goals of patient care.^12^ In other words, it is insufficient for the hospital to have protocols; it is also necessary to encourage the use of these protocols.

A national survey by Colleti et al. in 2020 reported that 57.1% of the physicians used formal sedation protocols.^26^ However, this result is not representative of the entire Brazilian territory, as most respondents were from the Southeast Region. This region is expected to follow more protocols, mainly because a greater number of centers of specialization and research are concentrated in this region. Another concern is that this survey was conducted only with physicians, but the state of sedation/agitation of the patient is directly or indirectly managed by a multidisciplinary team; therefore, it is important that all those involved in the team know and apply the sedation protocols.

Although the interest in validating sedation scales for the pediatric ICU has increased, which is inconsistent with the report by Amoretti et al.^15^ in 2008, some errors, such as the use of scales not adapted for children in the ICU, still occur. An example is the Ramsay scale, which, despite being one of the most commonly used scales in pediatric ICUs, has only been validated for deep sedation in children undergoing invasive procedures and only in English.^25,27^

The RASS has also been widely used in Brazilian pediatric ICUs for some time, but its first cross-cultural adaptation was only carried out in 2021.^17^ This scale is easy and quick to perform, which explains the high frequency of its use. However, the RASS is limited compared with the State Behavioral Scale regarding the dimensions included in its classification stages. Most of the items are restricted to the response to verbal commands, and ventilation is mentioned only in the agitated stage. The State Behavioral Scale, on the other hand, encompasses more domains at all of its sedation levels and provides richer descriptions for the classification of the behavioral state.

Notably, the State Behavioral Scale was originally designed for the pediatric population, which is important to consider because there are biobehavioral differences between adults and children. The scales most commonly used in Brazil were originally validated for the adult population; these scales were extrapolated to the pediatric population only after being validated in adults or have not yet gone through the validation process, as is the case with the Ramsay scale.^15,25^

The children in the present study were of similar ages to the children in the original study by Curley et al.^16^ but had longer ventilation and hospitalization times; these longer times were likely due to the characteristics of the ICUs of the hospital under study, where most patients were in the cardiac postoperative period and tended to have a more severe and prolonged evolution. However, even with prolonged periods of ventilation and hospitalization and, consequently, with a higher risk of withdrawal and delirium, a minority of patients had greater agitation scores (9.1% had a score of +1, and 2.3% had a score of +2).

During the application of the adapted scale, the majority of children (48.9%) had sedation scores of -1 and 0 on the State Behavioral Scale (corresponding to light sedation) according to the goals recommended in the current protocols. The study of the RASS adaptation also obtained results in agreement with good sedation practices (mean, -2.5, equivalent to light sedation), a positive result compared with the first study of cross-cultural adaptation of the COMFORT-B scale for pediatric patients, according to which 55% of the patients were highly sedated.^15,17^

These data highlight the importance of the increasing search for evaluations of the psychometric quality of sedation scales, improving from a lack of validated instruments for Brazilian children on mechanical ventilation to the presence of several validated scales. In addition, the search for objective, practical and easy-to-perform instruments has improved the adherence of multidisciplinary teams to protocols and sedation therapies guided by the goal of minimal sedation.

This study has several limitations. For example, this study was conducted in a single hospital in the city of Salvador, which may have resulted in sample bias. Although no significant semantic differences were found among the words contained in the instrument, studies involving other ICUs and other Brazilian states should be performed to confirm its applicability under different structural and cultural conditions, especially in large centers that offer more complex services. Another limitation was the use of a convenience sample, which reduces the generalizability of our results; thus, additional studies with larger and more diverse samples are needed.

This study was intended to stimulate new research, contribute to humane and high-quality care for patients in Brazilian pediatric ICUs and foster the desire for continuing and multidisciplinary education with critically ill children, who need comfort and safety during their stay in the pediatric ICU, as the center of care.

## CONCLUSION

The final adaptation of the State Behavioral Scale developed in this study was validated, revealing the quality of the items in representing the operational definitions in each domain. The psychometric properties of the adaptation indicate that it is representative of the sedation classification of children on mechanical ventilation.

The majority of patients were under minimal sedation, as recommended by the guidelines, confirming the importance of implementing sedation scales and protocols nationally; this result differs from data from studies performed prior to the use of validated scales for Brazilian children.
